# Keeping tabs on fructose

**DOI:** 10.7554/eLife.21263

**Published:** 2016-10-11

**Authors:** Anath Shalev

**Affiliations:** Comprehensive Diabetes Center, University of Alabama at Birmingham, Birmingham, United StatesShalev@uab.edu

**Keywords:** fructose, diabetes, metabolism, TXNIP, GLUT2, GLUT5, Mouse

## Abstract

Too much fructose in the diet can worsen metabolic problems via a process that involves thioredoxin-interacting protein.

**Related research article** Dotimas JR, Lee AW, Schmider AB, Carroll SH, Shah A, Bilen J, Elliott KR, Myers RB, Soberman RJ, Jun Yoshioka J, Lee RT. 2016. Diabetes regulates fructose absorption through thioredoxin-interacting protein. *eLife* **5**:e18313. doi: 10.7554/eLife.18313

Fructose is a simple sugar that is found in many fruits and plants. Its strong sweetness and minimal effect on blood glucose levels make fructose a more attractive sweetener than other naturally occurring sugars. As a result, high-fructose corn syrup is often added to a variety of foods and drinks to make them sweeter (Figure 1). This has lead to people consuming much more fructose than in previous decades, especially in the United States and other westernized countries (Cox, 2002; Goran et al., 2013). Along with this trend, more and more evidence suggests that consuming too much fructose could detrimentally affect our metabolism. In particular, excess fructose consumption has been linked to an increased risk of insulin resistance, obesity, type 2 diabetes and non-alcoholic fatty liver disease (Elliott et al., 2002; Kolderup and Svihus, 2015). However, it remains controversial whether the fructose itself actually causes these metabolic problems, and different studies have reported conflicting results (Campos and Tappy, 2016).

**Figure 1.**
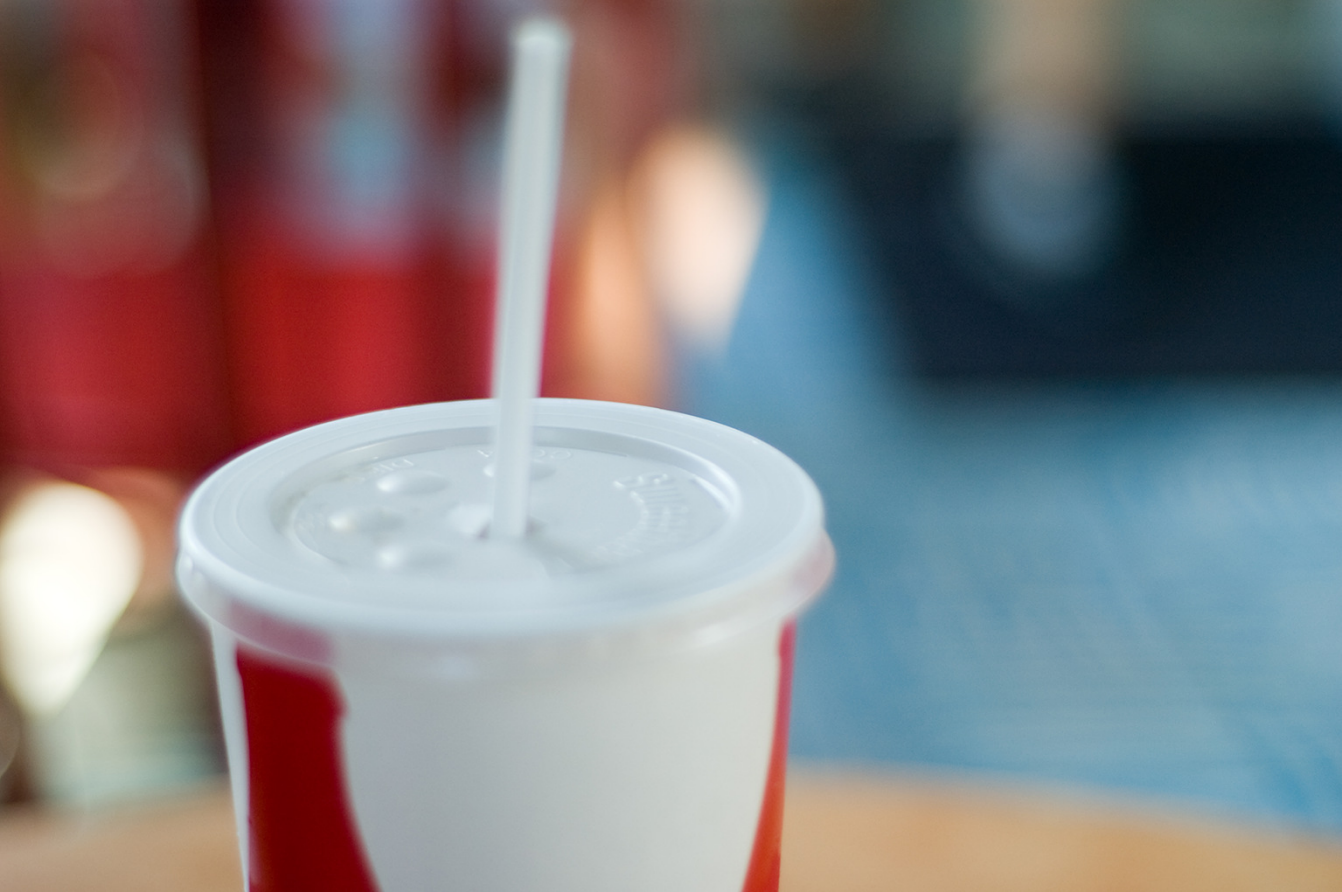
Fructose in food and drink. High fructose corn syrup – which is synthetically manufactured from broken down cornstarch – is added to many soft drinks to increase their sweetness, palatability and taste.

Image credit: “high fructose water color” by Laura Taylor (CC BY-NC-ND 2.0)

After we eat or drink fructose it is transported through the cells that line our small intestine with the help of sugar-transporting proteins called GLUT5 and GLUT2 (Gould et al., 1991; Burant et al., 1992). Once in the bloodstream, it is taken to the liver via the hepatic portal vein. The liver then removes some of the fructose in the blood; this ensures that fructose levels in the blood remain at least 10 times lower than glucose levels (Douard and Ferraris, 2008). However, the liver also converts fructose into a number of metabolites that can be used to increase stores of glucose and fat, and this might contribute to the detrimental effects on metabolism that are linked to eating fructose. The uptake of fructose by the small intestine is limited to control how much fructose gets into the blood and liver, but relatively little is known about this process.

Now, in eLife, Richard Lee and co-workers – including James Dotimas and Austin Lee as joint first authors – report that a protein referred to as TXNIP (which is short for thioredoxin-interacting protein) regulates fructose uptake via a previously unrecognized interaction with GLUT5 and GLUT2 (Dotimas et al., 2016). Normally, TXNIP acts to regulate the cell’s redox state. However, too much TXNIP can detrimentally affect how the body manages its glucose levels (referred to as glucose homeostasis) in a number of ways (Minn et al., 2005; Parikh et al., 2007; Chutkow et al., 2008; Xu et al., 2013).

The gene that encodes TXNIP is itself activated by sugars like glucose and fructose (Minn et al., 2005; Stoltzman et al., 2008; Cha-Molstad et al., 2009), and Dotimas et al. – who are based at Harvard and the Massachusetts General Hospital – confirmed that fructose promotes the production of TXNIP in the small intestine. They also went on to show that fructose actually promotes the interactions between TXNIP and GLUT5 and GLUT2 in the small intestine, and that TXNIP in turn increases fructose uptake.

By using mutant mice and radioactively labeled fructose, Dotimas et al. could show that mice fed fructose via a tube ended up with high levels of fructose in their blood and tissues, but only if they had a working copy of the gene for TXNIP. To confirm that TXNIP was making the small intestine absorb more fructose, they then performed a similar experiment but injected a solution of fructose directly into the bloodstream rather than feeding the mice via a tube. As expected, when the small intestine was bypassed like this, all the mice showed the same elevated levels of fructose in their tissues regardless of whether they had TXNIP or not (Dotimas et al., 2016).

Previous studies have shown that diabetes leads to increased production of TXNIP and that deleting the gene for TXNIP (or otherwise inhibiting the protein) can prevent diabetes, improve glucose tolerance and have a beneficial effect on glucose metabolism (Chen et al., 2008). Dotimas et al. found that mice without the gene for TXNIP were also protected against the detrimental effects of a high fructose diet on metabolism.

The researchers also found that triggering diabetes in mice (by killing their insulin-producing cells with a toxin called streptozotocin) led to more TXNIP being produced in the small intestine. This in turn resulted in more fructose being absorbed by the small intestine. Since deleting the gene for TXNIP diminished this effect, they propose that diabetes increases fructose absorption and that TXNIP is involved in this process. Indeed, the data show that TXNIP links fructose absorption to both glucose homeostasis and diabetes.

Though Dotimas et al. clearly demonstrate a new protein-protein interaction between TXNIP and the fructose transporters; it remains to be shown that this interaction actually causes the increase in fructose absorption. If indeed it does, the next challenge will be to work out exactly how this happens. Other challenges include determining how diabetes affects the levels of fructose circulating in the blood in humans, and to tease apart whether any changes in fructose levels are caused by the diabetes itself or by differences in diet.

In addition to supporting the notion that too much fructose in the diet is bad for metabolic control, at least in mice, the work of Lee, Dotimas, Lee and co-workers might also help explain why different studies have come to different conclusions and suggests that the context in which fructose is consumed is important. Just by itself – that is, without glucose being present and in the absence of diabetes or elevated TXNIP levels – very little fructose might be absorbed. In contrast, high levels of glucose will lead to an increase in TXNIP levels, which will promote the absorption of fructose and exacerbate existing problems with metabolism. In any case, the latest work is consistent with the overall concept that inhibiting TXNIP is beneficial for metabolism, and reveals yet another reason why this might be. Another interesting future research direction would be to ask how the gut microbiome might affect the way TXNIP regulates fructose uptake and any resulting metabolic sequelae or complications.
